# Metastatic Pancreatic Cancer Diagnosed From a Lesion in the External Auditory Canal

**DOI:** 10.7759/cureus.101632

**Published:** 2026-01-15

**Authors:** Louise Le Blevec

**Affiliations:** 1 Ear, Nose and Throat (ENT), Victoria Hospital Kirkcaldy, Kirkcaldy, GBR

**Keywords:** external auditory canal, external auditory canal lesion, metastasis, pancreatic adenocarcinoma, pancreatic cancer

## Abstract

Metastases to the external auditory canal (EAC) are extremely rare and most often arise from breast, renal or lung carcinoma. To our knowledge, this is the first case of metastatic pancreatic adenocarcinoma to the EAC. A 34-year-old male patient, a smoker, presented with a 15-month history of right otalgia, otorrhoea, and hearing loss. Examination revealed a large, fleshy, and friable EAC lesion suspicious for neoplastic aetiology. Biopsy reported poorly differentiated adenocarcinoma. Subsequent imaging revealed a large pancreatic head mass, confirming a diagnosis of metastatic pancreatic adenocarcinoma. Although metastatic tumours to the EAC are rare, the differential should be considered when managing patients with EAC lesions. Detailed assessment, including biopsy and imaging, should be undertaken promptly if the presentation is suspicious.

## Introduction

Pancreatic cancer is the twelfth most common cancer and the sixth highest cause of cancer mortality worldwide [1]. Pancreatic adenocarcinoma (PA) accounts for more than 90% of pancreatic tumours. PA typically presents in advanced stages, as early lesions are clinically silent, making early diagnosis challenging [2]. It commonly metastasises to regional nodes, the liver, adjacent organs and the lungs [3]. 

In this report, we present the case of a 34-year-old male smoker who exhibited a lesion in the external acoustic meatus (or ear canal) as the initial manifestation of metastatic pancreatic cancer. EAC cancers comprise <0.2% of head and neck malignancies, and metastatic involvement of the EAC is even rarer, with no reliably reported incidence [4]. On rare occasions, a lesion in the EAC may be the first indication of metastatic disease [5]. Cancers more commonly known to metastasise to the EAC include breast, renal and lung carcinoma [6]. Based on a PubMed, Embase, and Cochrane search using the terms "pancreas," "metastasis," and "external auditory canal," to our knowledge, this appears to be the first reported case of pancreatic adenocarcinoma metastasizing to the EAC.

## Case presentation

A male smoker in his 30s, with no significant past medical history, was referred to the Ear, Nose and Throat (ENT) clinic with a 15-month history of right-sided hearing loss and a five-month history of intermittent right-sided otalgia, accompanied by foul-smelling, blood-stained otorrhoea. He had been reviewed multiple times in the community and received sequential treatment for eustachian tube dysfunction, otitis externa and otitis media. Management included reassurance, topical ear drops, oral antibiotics and nasal sprays; however, none of these interventions proved effective. Due to worsening symptoms and clinical suspicion of cholesteatoma - based on the appearance of a white swelling overlying the tympanic membrane - he was urgently referred to ENT.

**Figure 1:**
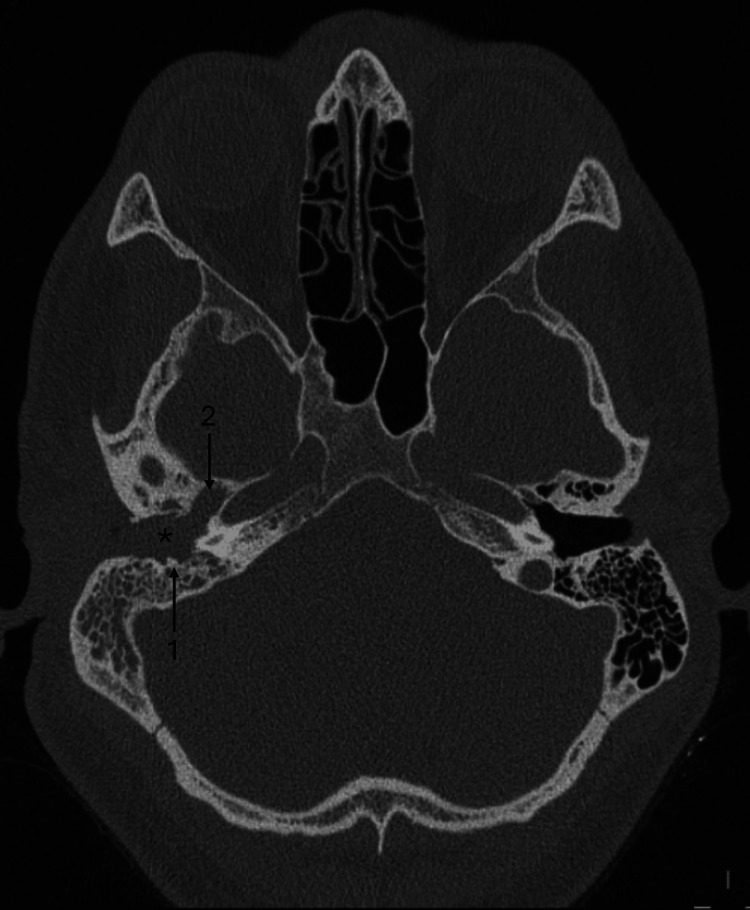
CT temporal bones CT temporal bones demonstrating a right EAC mass (*) causing erosion of the EAC (1) and opacification of the middle ear (2).

On examination, a large, fleshy, friable lesion was observed in the right ear canal containing hair follicles and appearing suspicious for neoplastic aetiology. No vertigo or facial palsy was noted. An in-clinic biopsy was inadequate but revealed concerning glandular epithelium with neoplastic features. Urgent CT of the temporal bones demonstrated extensive opacification of the right external auditory canal (EAC) and middle ear, with erosion of the canal walls, raising suspicion for malignancy (Figure 1). Additionally, fullness and apparent soft tissue thickening of the right nasopharynx were noted.

Examination under anaesthesia and biopsies were performed three weeks after the scan results. Once again, examination revealed a heaped, friable mass filling the right ear canal, which was highly haemorrhagic upon biopsy. A mass in the right post-nasal space had a similar macroscopic appearance to the ear lesion. Histopathological analysis of both the right ear lesion and post-nasal space biopsies revealed moderately to poorly differentiated adenocarcinoma. Immunophenotypically, the right ear lesion was positive for cytokeratin 7 (CK7) and negative for cytokeratin 20 (CK20), thyroid transcription factor-1 (TTF-1), caudal-type homeobox 2 (CDX2) and p16. Histopathological features were consistent with a hepatobiliary or upper gastrointestinal cancer.

**Figure 2:**
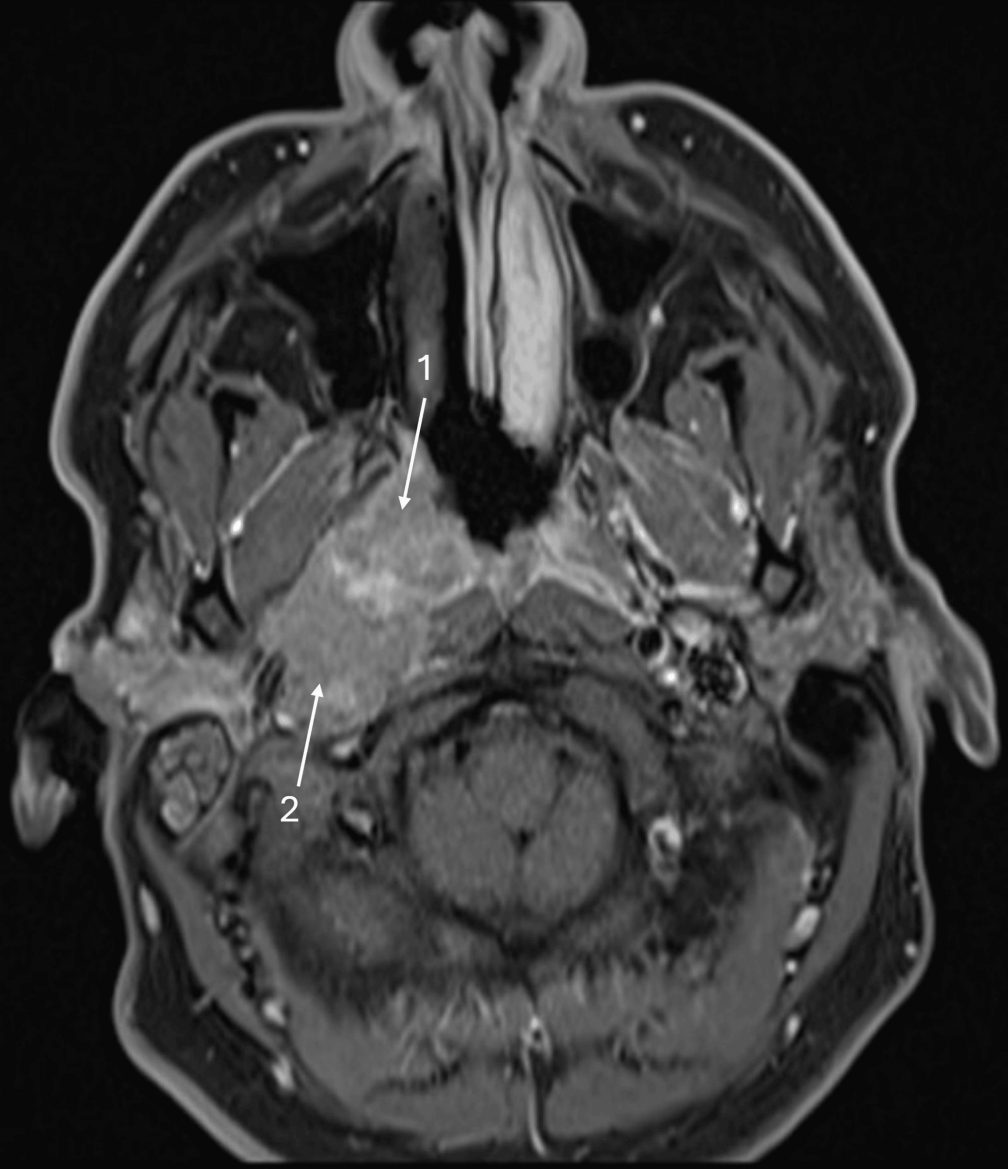
MRI internal acoustic meatus MRI internal acoustic meatus showing a right nasopharyngeal mass (1) extending into the right parapharyngeal space (2).

The patient was subsequently referred to the Head and Neck multidisciplinary team (MDT), whilst further imaging was undertaken, including a CT neck and chest, MRI of the internal acoustic meatus and neck, and an orthopantomogram. Imaging findings were consistent with an aggressive nasopharyngeal mass exhibiting soft tissue extension into the right parapharyngeal space (Figure 2), encasing and severely narrowing the right internal carotid artery. A separate enhancing, aggressive lesion was identified in the right EAC and middle ear cavity. Additionally, imaging confirmed the presence of suspicious bilateral cervical lymphadenopathy and small pulmonary nodules, raising concern for metastatic disease.

**Figure 3:**
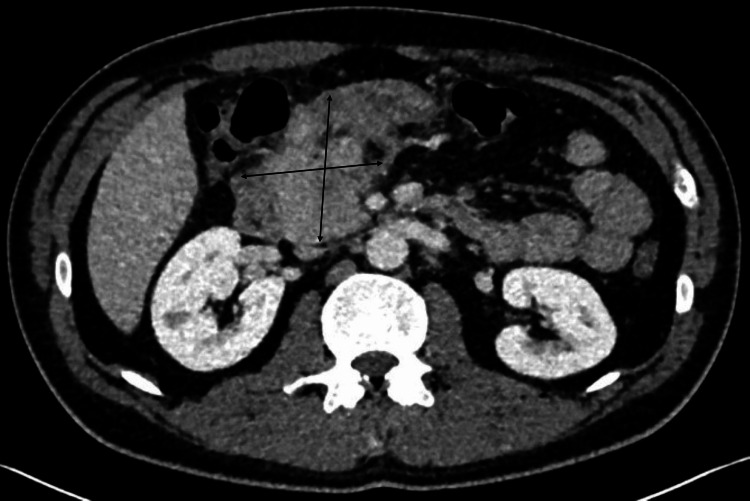
CT abdomen with contrast CT abdomen with contrast revealing a pancreatic head mass.

The Head and Neck MDT meeting took place two weeks later, during which completion staging with a contrast-enhanced CT scan of the abdomen and pelvis was planned. Cancer markers CA 19-9 were normal, and CEA was mildly raised (5.4 ug/L, normal range 0-5.0 ug/L). A referral was made to the Cancer of Unknown Primary (CUP) team, and a decision was reached to initiate palliative chemotherapy. On the same day, a CT of the abdomen and pelvis with contrast was performed, revealing a large mass in the pancreatic head (Figure 3) involving the portal vein with associated non-occlusive thrombosis. The scan also demonstrated probable local lymphadenopathy, a suspected left adrenal metastasis, and presumed omental infarction in the anterior upper abdomen.

The patient was subsequently reviewed by the Medical Oncology team, and a final diagnosis of metastatic pancreatic cancer was established, presenting with symptomatic metastases to the right EAC and parapharyngeal space. He was commenced on palliative chemotherapy with gemcitabine and abraxane. Unfortunately, he deteriorated and was switched to second-line palliative chemotherapy, but he did not respond to this. The patient passed away within six months of initial presentation to ENT.

## Discussion

There are multiple differential diagnoses for lesions of the EAC, including congenital (e.g., atresia), inflammatory (e.g., malignant otitis externa), benign (e.g., exostosis, osteoma and adenoma), and malignant lesions (e.g., squamous cell carcinoma, basal cell carcinomas and metastases). Symptoms of these overlap and are non-specific, including otorrhoea, otalgia, bleeding and hearing loss. This contributes to the clinically challenging diagnosis. Additionally, metastatic carcinoma to the EAC is extremely rare; cancers that commonly metastasise to this site include breast, renal and lung carcinoma [5-7]. Although this patient had no known primary malignancy or history of metastatic disease, a metastatic lesion in the EAC should not be forgotten as a differential diagnosis.

Given the wide range of potential causes for EAC lesions, early referral from primary care to specialist ENT services is recommended for persistent cases. Prompt biopsy and imaging should be considered by the specialist team to aid in accurate diagnosis. EAC malignancies are frequently misdiagnosed as otitis externa or otitis media, as occurred in this case, where multiple treatments were trialled prior to referral. We advocate for early ENT referral from primary care when patients present with refractory symptoms or symptoms that do not respond to standard treatment, as these may be indicative of carcinoma [8].

Pancreatic cancer remains one of the most challenging malignancies for both patients and clinicians due to its diagnostic complexity and poor prognosis. Approximately 90% of pancreatic cancers are adenocarcinomas or related subtypes. Although pancreatic adenocarcinoma has a relatively low incidence compared to other cancers, it carries a high mortality rate, with a five-year survival as low as 2% [2]. This poor prognosis is primarily due to late-stage presentation, with most cases diagnosed at locally advanced or metastatic stages, as seen in this patient. Contributing factors include the heterogeneous and non-specific nature of symptoms, the pancreas’s proximity to major blood vessels facilitating early spread, and the technical difficulty of surgical resection [3,9]. Metastatic spread to the EAC tends to be via vascular and/or perineural pathways [7]. In this context, the patient's EAC metastases can be presumed to be secondary to haematogenous spread.

Surgical resection remains the only potential curative option for pancreatic cancer; however, only about 20% of patients are eligible for curative surgery at the time of diagnosis [2,3]. Management of metastatic pancreatic cancer typically focuses on symptom control, treatment of jaundice, and palliative chemotherapy [8]. Pancreatic adenocarcinoma commonly metastasises to regional nodes, liver, adjacent organs and lungs [3]. To our knowledge, this is the first reported case of pancreatic cancer metastasising to the EAC, and as such, there is no standardised management protocol. Nonetheless, palliative chemotherapy remains the cornerstone of treatment for distant pancreatic metastases. Chemotherapy is generally favoured in the management of metastatic carcinoma to the EAC, regardless of the primary site, as these lesions most often result from distant metastases [7].

## Conclusions

Although metastatic tumours of the external auditory canal (EAC) are rare, they should be included in the differential diagnosis to facilitate timely identification and management. This case highlights a potentially delayed referral to ENT specialists, despite a history of symptoms persisting for over a year and failing to respond to various treatments. Therefore, early referral from primary care to specialist ENT services is strongly recommended for patients presenting with recurrent or refractory ear symptoms, as well as for any EAC lesions.

Pancreatic cancer is known for its heterogeneous presentation and non-specific symptoms, which pose significant challenges to early diagnosis and treatment. Consequently, a thorough evaluation of EAC lesions is essential. This should include prompt consideration of biopsy and imaging at the time of presentation, particularly when lesions exhibit suspicious features.
